# Supplementary material for: A beta-glucosidase of an insect herbivore determines both toxicity and deterrence of a dandelion defense metabolite
Source: eLife. 2021 Oct 11;10:e68642. doi: 10.7554/eLife.68642 (PMC8504966; doi:10.7554/eLife.68642)
Supplement: Supplementary file 1. [file elife-68642-supp1.docx]

**SUPPLEMENTARY FILE 1 Nuecleotide sequences of the heterologously expressed *M. melolontha* βglucosidases.**

>*Mm_bGlc1*

ATGAAGCATCTACTATTATTTTTAATTTTGGCGGTTTTTGCAAATGCGCAGAATTATTCATTCCCTGATGGTTTTCTATT

TGGTGTTTCTACAGCATCGTACCAGGTTGAGGGAGCTTGGAATATAAGTGGTAAGGGAGAAAATATATGGGACAGACTCG

TACATTCAACTCCAGAACGTGTAGTAGATATGAGTAATGGAGACGTAGCTTGCGATGCTTATCATAAAACAGAAAAAGAC

GTCCAGCTACTGAAAAACCTCAACGTAAACTTTTACAGATTTTCCATCTCCTGGTCCAGAATCCTCCCATCAGGTTACGT

TAACGTAATTAACCCCGATGGCATTCGATATTACAACGAATTAATTGACGAACTGTTGGCGAATGGTATTGAGCCCTTTG

TAACGATGTACCACTGGGATTTGCCCCAACCCCTGCAAGAAATCGGTGGCTGGGCTAATCCCTTAATATCCGATATATTT

GCTGATTATGCTGAACTTCTTTACTCGCAGTTCGGTGATAGGGTGAAAAATTGGATAACGTTTAACGAACCTCCTGTTGT

GTGTAGTGGGTATGCTGGAATTATGCCACCTGGGTATGATCAAGGAGGTATCGGTGATTATTTATGTGCACACCATCTGC

TTATTTCACACGGAAAGGCATATCGAATTTATGACGAAGAATACAGGAATGCTCAACAAGGCAGAGTTGGTATAACTATA

GATACTAGTTGGTATGAACCTGAATCAGATTCAGATTTGGACATTTCTGAAAGGGTACTGCAAATGAAGTATGGTCTATA

CGTACATCCAATTTTTTCCGAAACAGGGGATTATCCGCCGATACTAAGAAAAAGAGTGGACGATTTAAGTGTAGAACAAG

GTTACGCTCGTTCCAGACTACCTCATTTTACTCCAGATGAAATTGAATTCCTTCATGGTTCATCCGATTTCCTTGGTTTG

AACCATTACACATCTTATCTATGTTCTTCTTCTTCATACGATTTGTTACCTCCATCACAGTGGGCCGATACTGGTGCCGT

TTGTTATCAGTCAGACGAGTGGGAAAGTTCGTCTTCTACATGGTTGAAAGTCGTACCTTGGGGCTTTAGAAAGTTGCTAA

ACTGGATAAAAAATGAATACAACAATCCTGAAGTAATAATAACAGAAAACGGATTTTCTGATTCTCCTGGGGATGTTAAT

GATTGCAGAAGAGTTAATTATTATAATCAATACCTAGAAAGCCTGTTACAAGCAATTCATGAAGACGACTGTTACGTTAC

GGGTTACACAGCTTGGAGTTTAATGGATAATTTCGAATGGCTAATGGGTTATACCGCGAGATTTGGACTCTACATGGTCG

ATTTCGATGACGAAGACAGACCTAGAACGCCAAAAATGTCTTCGTTTATCTTCAAAAATATTATCGAAACTAGGCGAATT

GATTGGGATTACGCGCCGGAAGGATTCGAAGTTTGTGAATGG

> *Mm_bGlc2*

ATGAAACCAATCGGTTTAATTGTTTTAGTTTTCTTGGCCGCTGCCGACGCACAAAATTATTCCTTTCCCGATGATTTTAT

ATTCGGTGTTGCTACAGCGTCATATCAAGTTGAGGGAGCTTGGAATCTAAATGGTAAAGGAGAAAATATATGGGATAGAT

TGACGCATTCAACTCCAGAACTTATAGCAGATAACAGCAATGGAGACGTAGCCTGTGATACGTATCATAAAACTGAGGAT

GATGTGCAACTACTTAATAACATCGGAGTAAATTTCTACAGATTTTCCATATCGTGGTCCAGAATTCTTCCAACAGGTTA

CGTAAATGAAATTAATTCAGACGGAATTAGATATTATAACGAGTTAATCGATGAACTCTTGGCCAATGATATCCAGCCTC

TTGTTACGATGTTCCACTGGGATTTGCCCCAGCCTTTGCAGGAAATTGGTGGATGGACAACTCCTTTGATATCCGATTTA

TACGCTGATTATGCTGATATTCTTTATTCAGAGTTCGGTGATAGAGTGAAGAATTGGATAACCTTCAATGAGCCTAATGC

TGTTTGCGCTGGTTATGCTGGAAGCATGGCACCAGGGTATTATATAGAAGGCATAGGTGACTACTTATGCGCACATCATT

TGCTCATTTCTCATGGGAAAGCTTACCAAATTTACAATGAGAAATATAGGGATGCCCAACAAGGTATAGTTGGAATAACT

GTGTATACTCCATGGTATGAACCAGCTTCAGATTCAGAAGACGATATTCAAGCTGCTGAAAGAGCTATGCAAATGACGTA

TGGCATATACGTACATCCAATTTATTCAGAAACAGGTGATTATCCACCAGTATTGAGGGAAAGAGTAGATGCTTTAAGCG

CGGAACAAGGTTATGCGAGATCTAGATTGCCCTATTTTACCGAAGAGGAAATAGAACTCATTCGTGGCTCGTTTGATTTT

CTGGGTCTAAATCACTACACGACTTATTTATGCTATGCTGCCACTTACGATTTGCAACCTCCGTCACAATGGGGAGATAT

TGGTGCTGCCTACTACCAGTTAGATGAATGGGAAGGTTCTGGTTCTTCTTGGTTGAAGGTCGTACCTTGGGGTCTGAGAC

GTTTATTAAATTGGATTAAAAATGAATACAACAATCCCGAAGTGATAATAACAGAAAACGGAGTTTCTGATAACACTGGG

GATTTGAATGATTGCAGAAGAGTTTATTATTACAATCAATACTTGCAATCCGTTCTAGAAGCAATTTTTGAAGACGAATG

TGATGTAATAGGTTATACAGCTTGGAGTTTCGTAGACAATTTTGAATGGTTACAAGGTTATACTGAAAAATTCGGTCTTT

ATAGAGTTGATTTTGACGATGATGACAGGCCTAGAACTCCGAAAATGTCTTCTCATGTCTTCAGGAATATTATCGAAAGT

AGACAGATTGACTGGGATTACACACCAGATGGATTTGAAGCTTGCGATTGG

> *Mm_bGlc3*

ATGAAATCACTAATTTTAATTTTAGTTTTGGCGGTCTCAGTCAATGGGCAAATATACTCGTTTCCCGAAGACTTTCAATT

CGGCGTCGCTACAGCGTCCTATCAAGTTGAGGGAGCTTGGAATGTAAGCGGTAAAGGAGAAAACATATGGGACAGGTTAA

CACATTCAACTCCAGAACGTATCGTAGATAACAGTAATGGTGACGTAGCTTGTGATGCTTACCATAAAACCGCAGAAGAC

GTGCAACTGCTCAAAGACATCGGAGTCGATTTCTACAGATTTTCAATTTCATGGTCCAGAATCCTTCCAACCGGTTACGT

AAACGAAATTAATCCCGATGGCATTCGATATTACAACGAACTAATTGACGAACTATTGGCTAATAATATCGTACCTCTAG

TTACGATGTTCCATTGGGACTTGCCTCAGTCTTTGCAGGATATCGGTGGATGGCCAAATCCATTAATATCTGACCTATTC

GCCGATTATGCTGAAATTCTTTACTCACAGTTTGGCGACAGGGTGGCGAACTGGATAACGTTCAATGAACCTCCTGTTAT

TTGTGGTGGTTACGCGGGGGGTTTAGCGCCCGGGTATAATCAAGATGGCATAGGCGATTATTTATGCGCACACAATCTCC

TAATCTCACACGCAAGAGCGTATCGAATTTATGAAGAGAAATATAAGGACGTTCAAGAAGGTAGAGTTGGTATTACTGTG

AATACTAGATGGTACGAACCGGCTTCAGATTCAGCAGAAGATGCCGCAGCTGCCGAGAGAGCTATACAAATGATTTATGG

TATATACATACATCCAATTTATTCAGAAACAGGAGATTATCCACCAATATTAAGAGAAAGAGTAGATGCATTGAGTGCAG

AACAAGGTTACGCGAGATCTAGACTACCTTATTTTACAGAAGAAGAAATCGAACTCATTCGGGGTTCGTCCGACTTTTTG

GGTTTAAATCATTACACCAGTCTCTTATGTTCCTTTTCAACATACGATTTAAGTCCTCCATCTCAGTGGGCTGATACGGG

TGTAGCTTGTTACCAATCAGATGAGTGGGAAGGATCTGGTTCCTCTTGGTTAAAGGTTGTACCTTGGGGTCTGAGAAGTC

TCCTAAATTGGATCAAAAACGGATATAACAATCCCGAAGTCATAATAACGGAAAACGGAATTTCTGATAATACTGGAGAA

CTAAACGATTGTAGACGAATCAGCTATTATAATCAATATTTGGAAGCACTTTTGGAAGCTATTAATGAAGACGGATGTAA

TGTTAGTGGATACACAGCTTGGAGTTTCATGGATAATTTCGAATGGCTGCAGGGCTATACTGAAAGATTTGGACTTTACA

AAGTTGATTTCGATGATGATGATAGAACTAGAACTCCAAAAATGTCTTCTTACATCTTCAGGAATATTATTGAGAAAAGG

CAAATCGACTGGGACTATACACCGGATGGATTCGAAGTCTGCGAATGG

> *Mm_bGlc5*

ATGAAAGCGATTATTATATTGGCTTTACTGTGTTCTGTAACCAAAGCGCAAGATTACGAATTTCCCCAAGACTTTCTTTT

TGGTGTTGCTACAGCGTCTTATCAAATAGAAGGTGCTTGGAATGAAAGTGATAAAGGCGAAAATATATGGGATTATCTAA

CTCATAACGATCCAGATTTCGTGACTAATAGAGACAATGGTGATGACGCCTGTAAAGCTTACTATAAAACAGATGAGGAT

GTCCAAATGATTAAGGATGTAGGCGCTCATTTCTATAGATTTTCGCTGTCTTGGTCGAGACTCCTTCCAACAGGACACAT

TAATAAAGTCAGCGAAGATGGTGTTGCATATTATAACGATTTAATTGATCAATTATTAGCGAACGACATAATTCCATATG

TGACAATATTTCATTGGGATTTACCCCAGCCTTTGCTGGAGTTGGGCGGATGGCCTAATCCAGCTTTGGCTGATATCTAT

GCAGCTTACGCTAACTTCGTATTCAACGAATTTGGAGATAGAGTACAAAATTGGATTACCTTCAATGAACCGTACCAAAT

TTGTGAACAGGGTTTTTCGGACGGTAGCTTAGCACCTGGATATGCTCAACAAGGAATTGGCGGTTATTTATGTGGTCATA

CTGTTCTCCTAGCTCATTCGAAAGCTTATCATATCTATGACGACCTCTATAGGGAAACACAAGGAGGTAGAGTCGGTATA

GTCGTTCACGGTGCTTGGGGTGAACCAGAATCTGATTCAGATGAAGATGCACAAGCAGCTGAGAATTTCATTCAAATGAA

TTTCGGTTGGTTCTTACATCCAATTTACAGTGACGCTGGTGGATATCCACCCAGTATGGTTTCCGCTATACATGCACTAA

GTACCGAAGAAGGGTTTCCCAGATCAAGATTACCGCCTTTTACAGAAGAAGAGATCGCCAACCTAAAGGATACGTCGGAT

TTTCTGGGATTAAATCATTACGGAGCTTATTTATGCAGACCCCTCAGTGAGACAGACGAAGTGATATCACCTTCACATGT

TAAGGATATCGGAACTTATTATTATCTGTCCGAAGATTGGGATCAGAGTGCTTCGGATTGGTTTGCGGTGACACCTTGGG

GTTTGAGGAGCATGTTAAATTGGATCAAAGAAGAGTACGACAATCCCGAAGTAATCATTACAGAAAATGGTTTTACGGAT

ACTAGTGACGAGTTGCGTGATTGCGGTAGAGTTAATTACTATAATACATACTTGCAATCACTTCTGGAAGCCATACACGA

AGATGAATGCAGAGTTACCGGTTACACTGCTTGGAGTATCATGGACAACTTCGAATGGCAATTTGGTTACTCGGTGAAAT

TTGGAATGTACCACGTTGATTTCACCAATGAAGATAGGACCAGAACACCAAAAATGTCGTCATACGTATACAAGAATATA

ATTGAAACAAGGGCAATCGATACCGCCTTCGAAACTGACGATTTTGAAATGTGTGGGGCGTCCTCGTTGAATATGGCCAC

AGTAAATCAATTACTTGTAGGTCTTCTTGCCAGTTTGTACATTTATATTAGGCATTTT

> *Mm_bGlc6*

ATGAGACGCGTCTTAATATTAATCTTATTAGCGTGCCTGTGTGCGGCTCAAGATGAAGGGGAAGAAACAGATAACACTGT

GTTCCCAGATGACTTTCAGTTCGGTGTTGCCACAGCTGCTTATCAAACAGAAGGTGGTTGGGATGACGGTGGGAAAGGTG

AAAATATTTGGGACTACTTGCTCCACAATACCGATAATTTTACTGTCGATGGAAGTAGTGGCGATGATGCGTGCAAAAGT

TATTATAAAACTGAGGAAGATGTCGAATTATTGAAGAACATTGGTGTAAATTTCTATCGGTTCTCAATCTCGTGGTCTAG

AATTCTGCCGACTGGACATTCGTACAGCGTAAATCAAGTAGGCGTCAACTACTATAATGACTTGATTAACCGACTAGTAG

CTAATGGAATAGAACCAATGGTTACGATGTACCATTGGGATTTACCGCAACCCATGCAAGAATTGGGTGGTTGGCCCAAC

CCCCGTTTGGCAGAATATTTCGTAGATTATGCCGATGTACTCTTCAGACTTTTCGGAGACAGAGTCAAAACTTGGGTCAC

TATCAACGAACCGTATGAATTTTGCCATCGGGGATATGGAACGGGCGGGTTAGCACCTGGATACACGCAAGATGGAATTG

GAGATTATTTATGTGCTTATACTGTCCTTCTAGCTCATGCTAGAACTTATAGAATGTACCAGGAGAATTACTTAGAAGAT

CAAGGAGGAAGAATCGGAATATCCCTCAACAGTGATTGGTATGAAGGTGCAGATGTTAATGCTGAAGATACGGCGGCAGC

TGAAACCAGTAACCAAATGATGTTGGGCTGGTTCGCACACCCGATTTACCATGAAGATGGTGACTGGCCTGAAATAATGA

AAGAGAGAATTTATCAATTAAGTATGGACGCAGGATTTGTGAGAAGTCGACTTCCCACTTTCACTTCAGACGAAATAGAA

GAAATAAAAGGCAGTTATGACTTCTTTGGCCTTAATCACTATACCACACTTATTTGTACTCCAACTGATAGTGATAGTAA

CGAAATTGATGCCGAAATTGTGACTTCATATGAAAATGATGTTGGTACATCCTGTACTGTAAACGAGAATTGGGATGAAA

CGGCTAATGGATGGAGAGTCAATGCTGATGGTTTGCGAAGATTGTTGAATTTTATTAGTAGCGAGTACGGTAACCCGGAA

GTAATCATAACGGAAAATGGATACCCCGACGGTACAACTCGCGTTATAAATGACTGCAATAGAATTGATTATTACCACCA

ATACCTAGGAGCAGTACTGGAAGCAATATACGAGGATGGATGTAATGTTAAAGGATATGCAGCTTGGAGTTTATTGGATA

ACTTTGAATGGCAAATTGGCTATACGGTGAGGTTTGGTCTCCATTACGTAGAAGGGCGAATTCCAGCACACTGGCGGCCG

TTACTAGTGGATCCGAGCTCGGTACCAAGCTTGATGCATAGCTTGAGTATTCTAAAGGGCAATTCTGCAGATATCCAGCA

CAGTGGCGGCCGCTCGAGTCTAGAGGGCCCGCGGTTCTAA

> *Mm_bGlc11*

ATGAAAGTGCAGGTTGTATTAATTTTATGCATTTATTTATGTAATGCGCAAGAGTATGAATTCCCCGAAGGTTTTATATT

CGGCGTAGGTACCGCGTCATATCAAATAGAAGGTGCTTGGAATGAAAGCGATAAAGGAGAAAATATATGGGATTACCTAA

CTCATAACTATCCTGAATTTGTAATGGATAGCAGTAACGGTGACGTGGCAAGTGATGGTTATCACAAGCTAGATCAAGAT

ATCCAATTAATGAAGAATGTTGGTGTAGATTTCTATAGGTTTTCGCTTTCATGGTCGAGGATCCTTCCCACCGGTCACAT

AAATAAAGTCAGCGATGATGGTGTTCGCTATTACGATGAGCTAATCGATAAATTATTAGCGAATGACATACTTCCATACG

TAACGATATTTCATTGGGACTTACCTCAGCCCTTGTTGGAGATTGGTGGATGGCCTAACCCTGCTTTAGCCGACATTTTC

GCAAATTATGCCGATCTAGTGTTTCGTTTATTTGGAGATCGTGTTAAAAATTGGATCACTATTAACGAGCCGTATCAGAT

TTGCGAGGAAGGATTTTCAGAAGGGATATTGGCACCTGGTTATGCTCAACATGGAATCGGAGGTTACTTGTGCGGTCATA

CGGTATTGCTAGCCCATGCTAAAGCGTTTAAGATTTACAACGACAAGTACAGAGAGGAACAAGATGGCAGAGTTGGGATA

GTAGTTCATGGTGCGTGGGCTGAACCAAGTTCGGAATCAGAAGAGGACAAATCAGCTGCTGAAACATGGCAACAAATGAA

TTTTGGATGGTTTTTGCATCCAATTTACTCAGAATCTGGAGATTATCCATCAATTATGAAAGAAAGAATAGAAGATCTAA

GCGAATTAGAAGGATTTCCGCGATCCAGATTACCTGTATTCAGTCAGGAAGAGATCGAATTGCTAAAAGATTCTTCCGAT

TTCTTAGGTCTTAATCACTATGGATCCTTTCTATGTAAACCACTGGAGGAATCACAGATTATTCCATCACATGAGAATGA

TATTGGCAGCGAATGTTATTTATCGGATGATTGGGAGCCGAGCGCATCTCCATGGTTTAGCGTGACTCCGTGGGGTTTGA

GGAACATTCTAAATTGGATTAAGGAAGAATATGGTAATCCAGAAGTGATAATAACAGAAAGCGGTTTTACAGATTCGAGT

AATGACACACGTGATTGTGGAAGGGTTAATTATTATAACACTTACTTGGAATCATTGTTGGAAGCCATACACGAAGACGA

GTGCAACGTAAGCGGTTACACTGCTTGGAGCATTATCGATCTTTTTGAATGGCAATTTGGTTACACATATCGTTTTGGTA

TGCATTATGTGGATTTTGACGACCCAGATAGACCTAGAACGGCAAAAATGTCTTCCTATATTTACAAGAACATCATCGAA

ACGAGGAAAGTTGATTTGAGCTATGCCCCGGAAGAATTCGAAAAATGTGGAGCATCAGTTTTAATTGGAACGGCGGTGAA

CAAATTACTTATTGGCGTCTTTGGGATATTATTTATTCGTATGAGATATTTT

> *Mm_bGlc14*

ATGAGACGAATCATTTTCCTTTTGCTTCTAGGATGCTTATGCAAGGCACAGGATGGAGACCGCTTGTTTCCAGAAGACTT

TAAATTTGGCGTTGCCACAGCGTCTTATCAGATAGAAGGAGCATGGCAGGCTGACGGTAAAGGAGAGAATATCTGGGACT

ATCTAACCCACAACAAAGACACATTAATAGACGACGGCAGTAATGGCGACGTTGCATGCAATTCTTATAACAAAGTTGAT

GACGACGTGGAATTATTAGTTGAATTGAGAGTTCAATATTATAGATTCTCTATATCATGGTCTAGAATACTTCCAACCGG

TCATGCAAATGAAGTTAATGAGGCGGGAGTGCAATATTATAGTGACTTAATCGATAAACTTCTTGAAAATGGGATAACCC

CTTTGGTAACGATGTATCATTGGGATTTACCTCAACCGTTACAAGAAATTGGAGGCTGGACCAGCCCAGTACTAGTTGAC

CTCTTCGTAGATTATGCTAATGTTCTCTTTACAAGTTTTGGCGACAGAGTGACGAATTGGATTACTTTCAATGAACCCTA

TCAGATTTGTCAACAAGGATATTCAGAAGGCACTAAGGCACCGGCTTACACACAAGATGGTGTCGGTGGATATCTTTGTG

GTCATACTTTGCTGCTGGCCCATGCTTACACTTATCACCTTTATCACGATGACTATGCAGGAACGGATGGTAGAATTGGT

ATAACAGTTAATGGAGTGTGGGCTGAAGCAGCATCTGAAGATTCTGTAGATCAAACCGCTGCTGACGATTATATAGAATT

TCATTTTGGTTGGTTCTTTAATCCAATTTATATCGGAGACTATCCTCAAATCATGAAGGATAATATACAGGCCAGAAGTG

AGCTCGAAGGTTTTCCAGATACGAGATTACCTGAGTTCACCGAAACAGAAATTGCACTCTTACGTAGCTCTTCAGACTTT

CTCGGTCTAAACCATTACACAACCTATCAATGCACACCATTAGAAGATGTAGATTCGCTTGAACAACCTTCATTTAAAGT

AGACAGCGGCGTTAATTGCTGGTCACCTACTGATTGGGAAGGTGGTGCTTCATCTTGGTTGAAGGTCCATCCTTCCGGTC

TTACTAGCATATTGAATTGGATCAGGGAAAAATACGATAATCCCGAAGTTATAATAACTGAAAATGGTTTTTCTGAGGCT

GGTGAGGTGAATGACCTAAACGACTGTGATAGAGTTAGTTACTATAATGGATACTTATATGCCGTTTTGGATGCCTTGGA

AGATGGATGCCAAGTATCCGGGTATATGGCTTGGAGCCTCATGGATAACTTTGAATGGATGAGAGGCTACAGCGAACGAT

TTGGATTATACTATGTCGATTTCGAAGATGAAGACAGGCCCAGAACTGCAAAGAAATCTGCCCTCGTTTATAAAAATATA

ATTGAAACTAGAGCAATAGATTTGGATTATGATCCATCAGAATTGGGAACTTGTACTACTTCTGACGAGGACGAAGTGCC

AGAG

> *Mm_bGlc15*

ATGAAGCTCGTAATTTTCGCTCTGATTTTAACATACGTTAGCGCCCAAGATACTCGATTTCCCGATGGATTTAAATTTGG

GGTTGCTACGGCATCGTATCAGATAGAAGGCGCTTGGGATGCTAACGGTAAAGGAGAAAATATATGGGATAGGTTGACGC

ATACAAGACCTGAACTAATCGCAGACGGCAGCAATGGTGATATCGCATGCGATGCTTACCACAAAACTGAAGAAGACGTT

CAGTTGCTGAAAGGTCTGGGAGTTGATTTCTATCGTTTCTCCATCTCATGGTCGAGAATTCTTCCCACTGGTTATGCCGA

CGAAATAAATGAAGATGGTATTCGCTATTACAACGAATTAATTGATGAACTTCTGGCAAATGATATCACGCCATATGTGA

CAATGTATCATTGGGATTTGCCGCAGCCGTTGCAGGAGATAGGTGGCTGGCTAAACTCTTCTTTAGCTGATATTTTTGTT

GATTACGCAGATGTGCTTTATGAGCAATTCGGTGACAGAGTGAAAGATTGGATAACGTTTAATGAACCCATCCAGGTTTG

CGAAGCAGGTTATTCGAATGCTGGAAAAGCTCCAGCTTATACAATGGCAGGAGTTGGAGGTTATTTGTGTGGTCATACTT

TGCTTATTGCACATGGAAAGACTTATCGACTTTACAATGAAAAATATAGGGAAAGTCAACAAGGTAGGGTTGGTATAACT

ATCGATGCTGGTTGGTATGAACCAGTTTCGGACTCAGATACAGATATAGAAGCTGCCGAACGTAGCATGCAAATGAACTA

TGGTTGGTATGCACATCCAATCTTTTCTGAAACGGGGGATTACCCTGAAATTATGAAGGAAAGGGTGGCAGAATTAAGTG

AATTAGAAGGCTTCGAAACTTCTAGATTACCTTCCTTTACTCAAGAAGAAATCGAATTCATACGAGGCTCGTCCGACTTC

CTAGGTCTTAATCATTACAGTAGTTCCTTGTGTACATCGATACCAGAAGAATGGGCTTTAACTGGACCTAGTCAATGGAT

AGACGTTGGTAGTTTGTGCTTACCCAGTCCCGAGTGGGAAGTGGCTGCTTCTAGTTGGTTGTATGTTGTTCCTTGGGGAT

TAAGAAGACTGTTAAATTGGATTAAGAACGAATATGGCAATCCCGAGGTTATAATTACTGAGAATGGATTTTCTGATACC

GGTGAATTAAATGATTGTCGAAGAGTGAATTATTATAATTCATATTTAACGGCAGTTCTAGAGGCAATCTTAGAAGATGG

CTGCAACGTTTCAGGATATACGGCTTGGAGTTTTATGGATAATTTTGAATGGTTGATGGGTTACACGGAACGATTTGGAA

TTCACTATGTCGATTTCGAGGATCCCGACAGACCAAGAACTGCTAAAATGTCCGCTCATGTCTTTAAAAATATTATAGAA

ACAAGGGAAATCGATTGGAATTACACTCCAGATGGTTTCGAAGAGTGCGATTGGTCATAA

> *Mm_bGlc16*

ATGAAGGTTCTAGTTATACTTTGTACAGTAATATATTTATGTAAAGCCCAAGCTTACGAATTTCCAGACGATTTTCTATT

TGGCGTTGCTACGGCAGCTTATCAGATAGAAGGTGCTTGGGATACTAATGGAAAAGGAGAAAATATATGGGATAATTTGA

CGCATACCTATCCACACTTGGTTGTAGATGGTTCTAATGGAGATGTTGCATGCGATGCCTACAGTCATACTGCAGAAGAC

GTCCAACTGCTCAAAAATTTAGGCGTCGACTTTTATAGGTTTTCCTTCTCTTGGTCGAGAGTCTTACCCACTGGAAAGAC

TGATTACATAAACCCAGATGGAATACGCTATTATAATGAACTAATTGACACTTTATTAGAGAATAATATAGAACCTATGG

CCACGATGTATCACTTTGATTTACCACAACCTCTTGAAGACGAAGGAGGTTTCCTGAACATAGTCATCGCTGATTATTTC

GAAGATTACGCGGAAGTTCTGTACGAGAATTTTGGTGATCGCATCAAACAATGGATAACGTTTAATGAACCGTCTCCTAT

ATGTGAAAATGGATACGGCGCAGACAGTATGGCACCTTTGGCAAATCAACCGGGAGTTGGTGGTTATATCTGTAGTCGAA

CTTTATTAGTGGCCCATGCTAAAGCTTATCATCTCTACAATGATCGTTACAAGGGAAGTCAAGGAGGTCGAGTGGGTATT

ACTATAAACAACTTTTGGTATGAACCTTATGCTGATACTACAGACTCAGCAGTAGAGTTAGCATTACAGGTTGCGTTTGG

TTGGTTTACTCATCCCATATATTCTAAAGAAGGCGATTTCCCACCAGCCATGAAAGAAAGAATAGCTAGATTAAGTGAAG

AGGAAGGCTTTTCTGCATCCAGACTACCAGAACTTACAACGGAAGAAATAGAATTAATAAAAGGATCATCTGACTTTTTC

GGTTTGAATCACTACACCACTCACTTCTGTTCCGAGTCTGGTATCGACTCGATATCACGACCATCCCATAATTACGACAT

GGGTGTAACATGCATGCCAGATTATAGTTACGAAGTCGCCGGATCGTTTTGGTTGAGCGTTATTCCTTGGGGGTTGAGGA

AGATGCTTAATTGGATCAAAGAAGAATACAACAATCCGGAAATTATAATCACTGAAAATGGATACTCCGATAATGAAGTT

ATTTTGAACGATTGCCGCAGAATTAATTACTATAATTCTTATTTAACAGAATTGTTAAATGCTATTTACGAAGATAACTG

TAACGTCACTGGTTACACAGCGTGGAGTTTTATGGATAATTTTGAATGGAGAATGGGTTATAGCGAAAAATTTGGTTTAT

ACTCAGTAGATTTTAATGATCCCGATAGGACAAGGACAGCTAAAATGTCAGCATACATATACAAAAATATTATTGAAACA

AGGCAAATTGACTGGAGTTATGTGCCCGAAAATTTTAGCGACTGTGAATGG

> *Mm_bGlc17*

ATGAAGAGACTAATTCTTATTTTCGTATTGGCTTGTTTAGCCAAAGCACAAGATTATCAATTTCCTGAAGGGTTTAAATT

TGGTGTCGCTACAGCCTCGTATCAAGTGGAGGGCGCTTGGAACGAAAATGGCAAAGGCGAAAACATATGGGACAGATTAA

CACACACCCAACCTGATTTAATCGCTGATAATAGCAATGGTGACATCGCTTGTGATGCCTACCACAAAACCGAAGAAGAC

GTTCAATTATTAAAGAACCTTGGAGTCAATTTCTACCGTTTCTCGATATCGTGGTCGAGAATTCTTCCAACAGGTTACAC

GAATGTCGTGAACGAAGATGGTATTCGATACTATAACGCATTAATCGACGCTCTTTTGGAAAACGGTATTACTCCGCTTG

TAACGATGTTCCATTGGGATTTACCACAGCCGTTGCAAGAAATCGGTGGCTGGCCCAATCCGCTTCTGGCTGATATATTT

GCAGATTATGCTGACATACTTTATCGAGAATTCGGAGATAGGGTGAAGGATTGGCTAACTTTCAACGAACCCACTCCCAT

TTGTATTGGAGGTTATTCGGAGGGTTGGATGGCGCCGGCTTATGAATTACAAGGTGTTGGAGGTTACCTGTGTGGTCATA

CATTACTTATTGCTCATGGGAAAGCTTATCGGCTTTACAACGAAAAGTATAGAGATACTCAGGAAGGTAGAGTTGGTATA

ACAATTGACAGCGGTTGGTATGAACCTGCTTCAGAATCAGAAGAAGACATTCAAGCAGCTGAAGACAGTGTACATATAAA

GTACGGTTGGATGGTCCATCCGATCTATTCAGAGACCGGTGATTATCCCCCTGTATTGAGAGAAAGGGTAGACGCATTGA

GTGCTGAAGAAGGCTTTGCGAGGTCCAGATTGCCTATCTTTACGGAGGAGGAAATCGAACTTATTAAAGGCTCTAGTGAT

TTTCTGGGTTTGAATCACTATACTACCAATCTCTGCACCCCCATTCCGGAAGAATGGGGTGTGGTGGGTCCTTCACATTA

CGTAGATAGTGGTGCTAATTGTTACCAAGATCCCTCGTGGGAAGGTTCGGGCTCGTCTTGGTTAAAGGTTGTACCTTGGG

GTTTGAGGAGATTGTTGAATTGGATTAAGGAAAATTACGACAATCCCGAAGTATTAATAACGGAGAATGGAGTTTCTGAT

AATACTGGTATTTTGAATGATTGCAGGAGGATCAATTTCTATAACACATATTTAACGGCAGTTCTTGAAGCTATTCACGT

GGACGGTTGCAATGTGGTTGGATATACAGCTTGGAGTTTCATGGACAACTTCGAATGGATGCAGGGATATACTGAACGAT

TTGGACTTTACCACGTCGATTTTAACGACACCGACAGGACGAGAACTCCAAAAATGTCATCCCATGTTTACAGACACATT

ATTGAGACTAAGCAAATAGACTGGGATTATACGCCTCATGGATTTGATGCCTGCGAGTGG

> *Mm_bGlc18*

ATGGGATACTTTGAACCATTAATAGTTTTCTTGATATCTTTATGGCGAGCCCAGAGTTATGTTTTTCCGGAAGAATTTTT

GTTTGGTGTTGCTACGTCCACGTATCAAGTCGAAGGTGCCTGGAATCTAAGTGGTAAAGGTGTAAATATATGGGACCACT

TGACCCATACAAATCCTGAGTTCACGGCGGACGGCAGTAATGGTGATGTAGCTTGCGATGCCTATCACAAAACTAAAGAA

GATGTCCAACTTATGGTGGATATGGGTCTTGACGTTTATCGTTTTTCCCTATCATGGACGAGAATTCTTCCAAACGGTTA

CAATAATTACACGAATCCCGATGGTGTTCGCTACTATAACGAATTGATCGATACTTTATTGGCGAATGGCATAAAGCCAT

TAGTCACGATGTTTCATTGGGACATACCGCAAGTATTTCAAGACGATTACGGTGGATGGTTAGGCTCGGATATGGTAGAT

ATTTTCGTCGATTATGCTGATGTCGCTTTTTCGTTGTTTGGCGATAGAGTGAAGGATTGGATTACTTTTAATGAACTTCA

TATATTTTGTGAACTTGGACATTCCATGGATATTATGGCACCGGCTCTTGGATTGAGCGGAGTTGGTGGTTATTTATGCG

CCCATAATATATTAATAGCTCATGGAAAGACTTATAAATTGTACAATGAGAAGTACAGGGATATTCAGAAAGGTAGAATT

GGTATCACAATTGATGGTGAATGGAAAGAACCGGCATCCACTTGTCCTGAAGATATAGAAACGGCAGAACGAGCTCTACA

AATGGAGTTTGGTTGGATAGCGCACCCAATCTTCTCAGAATCCGGCGATTATCCACCCGTTATGAGGAGCAGAATTGACG

TGATGAGTGCCGAAGAAGGTCTGAAGACATCAAGATTGCCCTATTTTTCCAAAGAGGAGATCGAATTAATAAGAGGATCG

GCTGATTTCCTAGGTTTAAATCACTACACGGCTTCGTTGTGTTCGTCAGAATTCAAAGAGTTACCAAGCAGGCCTTCGTA

TACTAGTGACACTGGAGCTAATTGTTATCAACCCGATTATTGGGAACCTACTGGCGTGTCGCAATTTAAAGTTACGCCAT

GGGCTTTTGGAAAGTTGTTGGTTTGGATCAAAAACGAATATAACAATCCGGAAGTTATCGTTACAGAGAATGGATATTCT

GATAATACGGGTGATTTATATGATTGTAGAAGAGTATATTATTACAATTCTTACCTTACTGAACTATTACATGTTGTTAA

CGATGAAGGATGTAGGATTACTGGTTACATGGCTTGGAGTTTCATGGATAATTTCGAATGGGGTAATGGTTACACAGCTA

AATTTGGGATTGTTAACGTCGACTTTAATGATGCTGACAGACCGAGAACTCACAAGATGTCCTCGTTCGTTTACAGAAAT

ATAATCCAGACGAGAGCGATCGATTGGAACTTTACTCCAGAGGGATTTGAAGCATGTTCGTGGTGG

> *Mm_bGlc19v*

ATGTCGTCATATGTATACAAAAACATTATAGAAACTAAAACAATCGACGAAGATTTTGTACCTGAAGGATTTGCTGCTTG

CAGTTCTAGTGAATACACCTTCCCTGACGGTTTCATATTTGGTGTCGCTACTTCAGCTTATCAAGTGGAAGGTGCTTGGG

AAGATGATGGAAAGGGAGAGAATATCTGGGACCATTTAACACACACAAGACCGACCGCCATAACTGATGAGAGTAACGGT

GACATCGCTTGCGACACGTATCACAAGACGGTGGAAGATGTTCAGTTACTAAACACATTGGGCGTAGATTTTTACCGTTT

TTCACTATCTTGGTCAAGACTTCTCCCTACCGGTCACGCCAACACTGTAAATCCTTTAGGCGTTGCTTACTATAACGAAT

TAATAGACGAGTTGATAGCAAATGATATAACACCATTGGTTACACTATTTCATTGGGATCTGCCACAACCACTGCAAGAA

ATTGGCGGTTGGCCTAATCCACTTCTTGTGGATTTATATGCCGATTATGCTAACATTGTTTTCAGTCTGTTTGGTGATCG

CGTTAAAGATTGGATTACGTTCAATGAGCCTTATCAGATATGTCAAGAGGCTTATTCGGAGGCAAATAAAGCTCCTGCAT

ACAATCAAGATGGCACTGGTGGCTATTTGTGCGCTTACACAGTATTACTTGCACATGCAAGAGCGTATCGTTTGTACGAG

ACAACATATAAGGAAGCACAACAAGGTAGAGTTGGAATTACCGTGCACGGAATATGGGCAGAACCTGAAACCGCAACTGA

TGCTGATATAGCTGCCGCTGAAAGTTATCAACAATTCCACTTTGGTATATATCTTCATCCAATATTCTCAGAAGAGGGCG

ATTTTCCGGAAATTGTAAAAACTAGAGTGAAGCAGATAAGTGACGTCATAGGCTACTCCAACAATCGCTTGCCAGTATTC

ACGGCTGAAGAAATCGAGTATATCCATGGTACCTCCGATTTCCTGGGCTTCAATCATTATTCGACAGACCTATGTAAAGC

AGCTGACGACGCTTCGTTAGTGCATCCATCCAATAAGGGTGATACTGGGGCTGATTGCCAAAAGTCCGATGACTGGGAAA

GCGCCGCTTCTTCGTGGGTGAAAGTTGTTCCGTGGGGTTTCAGAAAATTGTTAAACTGGATTAAAGACGAATATAATAAT

CCAGAAGTACTGATTACTGAAAATGGTTTCTCCACATTTGGTGATGATTTGAACGACTGCAGAAGAATTAACTACTTCAA

CGAATATCTAACCGCCCTTTTGGAAGCAATACACGAGGACGGATGCAACGTTATAGGATACACAGCTTGGAGCTTCCTTG

ATAATTTCGAATGGATGGATGGTTATTTAGAGAAGTTCGGTTTATACATGGTCGACTTTGACGATCCAGATAGACCGAGA

CAACCAAAAATGTCTTCCTATGTTTACACAAATATCATCCAGACACGAAGAGTCGACACGAGCTTCACCCCTGAAGGTTT

TGAAGCTTGCATATTTGATGAAGATACCGAGTCAGATGAAGATGTTTAA
