# Supplementary material for: A beta-glucosidase of an insect herbivore determines both toxicity and deterrence of a dandelion defense metabolite
Source: eLife. 2021 Oct 11;10:e68642. doi: 10.7554/eLife.68642 (PMC8504966; doi:10.7554/eLife.68642)
Supplement: Supplementary file 2. [file elife-68642-supp2.docx]

**SUPPLEMENTARY FILE 2 Primer information.**

| **Primer name** | **Primer sequences (5‘ -> 3‘)** | | | **Target gene** | | |  |
| --- | --- | --- | --- | --- | --- | --- | --- |
| Primers to amplify full-length seuqences of *M. melolontha* β-glucosidases. | | | | | | |  |
| 1-pIB-fwd | GTAATGAAGCATCTACTATTATTTTTAAT | | | Mm_bGlc1 | | |  |
| 1-pIB-rev | CCATTCACAAACTTCGAATCCTTC | | | Mm_bGlc1 | | |  |
| 2-pIB-fwd | GTAATGAAACCAATCGGTTTAATTGTT | | | Mm_bGlc2 | | |  |
| 2-pIB-rev | CCAATCGCAAGCTTCAAATCCAT | | | Mm_bGlc2 | | |  |
| 3(2)-pIB-fwd | GTAATGAAATCACTAATTTTAATTTTAGTT | | | Mm_bGlc3 | | |  |
| 3(2)-pIB-rev | CCATTCGCAGACTTCGAATCCAT | | | Mm_bGlc3 | | |  |
| 5-pIB-fwd | GATATGAAAGCGATTATTATATTGGCT | | | Mm_bGlc5 | | |  |
| 5-pIB-rev | AAAATGCCTAATATAAATGTACAAAC | | | Mm_bGlc5 | | |  |
| 6neu-pIB-fwd | GTAATGAGGCGCGTCTTAATATTAATC | | | Mm_bGlc6 | | |  |
| 6neu-pIB-rev | TAGAATACTCAAGCTATGCATCAAG | | | Mm_bGlc6 | | |  |
| 11-pIB-fwd | GATATGAAAGTGCAGGTTGTATTAAT | | | Mm_bGlc11 | | |  |
| 11-pIB-rev | AAAATATCTCATACGAATAAATAATA | | | Mm_bGlc11 | | |  |
| 14-pIB-fwd | GATATGAGACGAATCATTTTCCTTTTG | | | Mm_bGlc14 | | |  |
| 14-pIB-rev | CTCTGGCACTTCGTCCTCGTCA | | | Mm_bGlc14 | | |  |
| 15-pIB-fwd | GTA ATGAAGCTCGTAATTTTCGCTCTG | | | Mm_bGlc15 | | |  |
| 15-pIB-rev | TGACCAATCGCACTCTTCGAAACC | | | Mm_bGlc15 | | |  |
| 16-pIB-fwd | GATATGAAGGTTCTAGTTATACTTTG | | | Mm_bGlc16 | | |  |
| 16-pIB-rev | CCATTCACAGTCGCTAAAATTTT | | | Mm_bGlc16 | | |  |
| 17-pIB-fwd | GATATGAAGAGACTAATTCTTATTTTC | | | Mm_bGlc17 | | |  |
| 17-pIB-rev | CCACTCGCAGGCATCAAATCCA | | | Mm_bGlc17 | | |  |
| 18-pIB-fwd | GATATGGGATACTTTGAACCATTAATA | | | Mm_bGlc18 | | |  |
| 18-pIB-rev | CCACCACGAACATGCTTCAAAT | | | Mm_bGlc18 | | |  |
| 19v-pIB-fwd | GATATGTCGTCATATGTATACAAAAAC | | | Mm_bGlc19v | | |  |
| 19v-pIB-rev | AACATCTTCATCTGACTCGGTA | | | Mm_bGlc19v | | |  |
| 5‘ RACE and nested-PCR primer | | | | | | |  |
| Mm_6-5’-RACE | CAATTTCGGCATCAATTTCGTTACTATCACTA | | | Mm_bGlc6 | | |  |
| Mm_6-5’-Nest | CACAAATCCTGCGTCCATACTTAATTGATA | | | Mm_bGlc6 | | |  |
| 3‘ RACE und Nested-PCR Primer | | | | | | |  |
| Mm_15-3’-RACE | | | | CTGATACCGGTGAATTAAATGATTGTCGAAGAG | | Mm_bGlc15 | |
| Mm_15-3’-Nest | | | | CAGTTCTAGAGGCAATCGTAGAAGATG | | Mm_bGlc15 | |
| Mm_6-3’-RACE | | | | TTATACTGTCCTTCTAGCTCATGCTAGAAC | | Mm_bGlc6 | |
| Mm_6-3’-Nest | | | | TGCAGATGTTAATGCTGAAGATACGGC | | Mm_bGlc6 | |
| SMARTer RACE cDNA Amplification Kit Primer | | | | | | |  |
| Long Universal Primer | | | CTAATACGACTCACTATAGGGCAAGCAGTGGTATCAACGCAGAGT | | | |  |
| Short Universal Primer | | | CTAATACGACTCACTATAGGGC | | | |  |
| Nested Universal Primer | | | AAGCAGTGGTATCAACGCAGAGT | | | |  |
| Primers for dsRNA biosynthesis with and without the T7 promoter sequence (highlighted in green). | | | | | | |  |

| Mm-Tubulin-fwd  Mm-Tubulin-rev  GFP-RNAi_fwd | CACGCATACGACTTTGGA  GAGGCCGTGATTGAAGAT  GCTTTGCAAGATACCCAG |
| --- | --- |
| GFP-RNAi_rev | GTTCATCCATGCCATGTG |
| Mm_bGlc_16_fwd_T7 | TAATACGACTCACTATAGGGAGAACAGTATGGCACCTTTGGC |
| Mm_bGlc_16_rev_T7 | TAATACGACTCACTATAGGGAGATCTAGCTATTCTTTCTTTCATGG |
| Mm_bGlc_16_fwd | ACAGTATGGCACCTTTGGC |
| Mm_bGlc_16_rev | TCTAGCTATTCTTTCTTTCATGG |
| Mm_bGlc_17_fwd_T7 | TAATACGACTCACTATAGGGAGAACCCACTCCCATTTGTATTGG |
| Mm_bGlc_17_rev_T7 | TAATACGACTCACTATAGGGAGAGATAGGCAATCTGGACCTCG |
| Mm_bGlc_17_fwd | ACCCACTCCCATTTGTATTGG |
| Mm_bGlc_17_rev | GATAGGCAATCTGGACCTCG |
| Mm_bGlc_18_fwd_T7 | TAATACGACTCACTATAGGGAGATTCATATATTTTGTGAACTTGGAC |
| Mm_bGlc_18_rev_T7 | TAATACGACTCACTATAGGGAGAGTCTTCAGACCTTCTTCGG |
| Mm_bGlc_18_fwd | TTCATATATTTTGTGAACTTGGAC |
| Mm_bGlc_18_rev | GTCTTCAGACCTTCTTCGG |
| Primers for RT-qPCR | |
| qPCR_Mm _bGlc_16_fwd | GACTCAGCAGTAGAGTTAGCATTACAGG |
| qPCR_Mm _bGlc_16_rev | CGTTGTAAGTTCTGGTAGTCTGGATGC |
| qPCR_Mm _bGlc_17_fwd | GAAGACATTCAAGCAGCTGAAGACAGTG |
| qPCR_Mm _bGlc_17_rev | CCTCCGTAAAGATAGGCAATCTGGAC |
| qPCR_Mm _bGlc_18_fwd | TACAAATGGAGTTTGGTTGGATAGCG |
| qPCR_Mm _bGlc_18_rev | GGCACTCATCACGTCAATTCTGC |
| qPCR_Mm _actin-fwd | CTGCCAGCTCAAGTTCCC |
| qPCR_Mm _actin-rev | GAACAGAGCTTCTGGGCA |
| qPCR_Mm_Tubulin_fwd | CACGCATACGACTTTGGAACAC |
| qPCR_Mm_Tubulin_rev | GAGGCCGTGATTGAAGATACG |
