# Supplementary material for: A beta-glucosidase of an insect herbivore determines both toxicity and deterrence of a dandelion defense metabolite
Source: eLife. 2021 Oct 11;10:e68642. doi: 10.7554/eLife.68642 (PMC8504966; doi:10.7554/eLife.68642)
Supplement: Supplementary file 3. [file elife-68642-supp3.docx]

**SUPPLEMENTARY FILE 3** Fragment nucleotide sequence of *tubulin*, *GFP*, *Mm_bGlc16*, *Mm_bGlc17*, *Mm_bGlc18* in *pCR®2.1-TOPO® and pIB/V5-His-TOPO®* vectors. The T7-Promotor sequence for *in vitro* RNA synthesis are highlighted in yellow.

Tubulin forward in pCR®2.1-TOPO®

GTT CAT CCA TGC CAT GTG TAA TCC CAG CAG CTG TTA CAA ACT CAA GAA GGA CCA TGT GGT CTC TCT TTT CGT TGG GAT CTT TCG AAA GGG CAG ATT GTG TGG ACA GGT AAT GGT TGT CTG GTA AAA GGA CAG GGC CAT CGC CAA TTG GAG TAT TTT GTT GAT AAT GGT CTG CTA GTT GAA CGC TTC CAT CTT CAA TGT TGT GTC TGG TTT TGA AGT TAA CTT TGA TTC CAT TCT TTT GTT TGT CTG CAG TGA TGT AGA CCT TGT GGC TGT TGT AGT TGT ATT CCA ACT TGT GGC CGA GGA TGT TTC CGT CCT CCT TGA AAT CGA TTC CCT TAA GCT CGA TCC TGT TGA CGA GGG TGT CTC CCT CAA ACT TGA CTT CAG CAC GTG TCT TGT AGT TCC CGT CGT CCT TGA AGA AGA TGG TCC TCT CCT GTA CGT ATC CCT CAG GCA TGG CGC TCT TGA AGA AGT CGT GCT GCT TCA TAT GAT CTG GGT ATC TTG CAA AGC

Tubulin rev in pCR®2.1-TOPO®

GCT TTG CAA GAT ACC CAG ATC ATA TGA AGC AGC ACG ACT TCT TCA AGA GCG CCA TGC CTG AGG GAT ACG TAC AGG AGA GGA CCA TCT TCT TCA AGG ACG ACG GGA ACT ACA AGA CAC GTG CTG AAG TCA AGT TTG AGG GAG ACA CCC TCG TCA ACA GGA TCG AGC TTA AGG GAA TCG ATT TCA AGG AGG ACG GAA ACA TCC TCG GCC ACA AGT TGG AAT ACA ACT ACA ACA GCC ACA AGG TCT ACA TCA CTG CAG ACA AAC AAA AGA ATG GAA TCA AAG TTA ACT TCA AAA CCA GAC ACA ACA TTG AAG ATG GAA GCG TTC AAC TAG CAG ACC ATT ATC AAC AAA ATA CTC CAA TTG GCG ATG GCC CTG TCC TTT TAC CAG ACA ACC ATT ACC TGT CCA CAC AAT CTG CCC TTT CGA AAG ATC CCA ACG AAA AGA GAG ACC ACA TGG TCC TTC TTG AGT TTG TAA CAG CTG CTG GGA TTA CAC ATG GCA TGG ATG AAC

GFP forward in pCR®2.1-TOPO®

GTT CAT CCA TGC CAT GTG TAA TCC CAG CAG CTG TTA CAA ACT CAA GAA GGA CCA TGT GGT CTC TCT TTT CGT TGG GAT CTT TCG AAA GGG CAG ATT GTG TGG ACA GGT AAT GGT TGT CTG GTA AAA GGA CAG GGC CAT CGC CAA TTG GAG TAT TTT GTT GAT AAT GGT CTG CTA GTT GAA CGC TTC CAT CTT CAA TGT TGT GTC TGG TTT TGA AGT TAA CTT TGA TTC CAT TCT TTT GTT TGT CTG CAG TGA TGT AGA CCT TGT GGC TGT TGT AGT TGT ATT CCA ACT TGT GGC CGA GGA TGT TTC CGT CCT CCT TGA AAT CGA TTC CCT TAA GCT CGA TCC TGT TGA CGA GGG TGT CTC CCT CAA ACT TGA CTT CAG CAC GTG TCT TGT AGT TCC CGT CGT CCT TGA AGA AGA TGG TCC TCT CCT GTA CGT ATC CCT CAG GCA TGG CGC TCT TGA AGA AGT CGT GCT GCT TCA TAT GAT CTG GGT ATC TTG CAA AGC

GFP rev in pCR®2.1-TOPO®

GCT TTG CAA GAT ACC CAG ATC ATA TGA AGC AGC ACG ACT TCT TCA AGA GCG CCA TGC CTG AGG GAT ACG TAC AGG AGA GGA CCA TCT TCT TCA AGG ACG ACG GGA ACT ACA AGA CAC GTG CTG AAG TCA AGT TTG AGG GAG ACA CCC TCG TCA ACA GGA TCG AGC TTA AGG GAA TCG ATT TCA AGG AGG ACG GAA ACA TCC TCG GCC ACA AGT TGG AAT ACA ACT ACA ACA GCC ACA AGG TCT ACA TCA CTG CAG ACA AAC AAA AGA ATG GAA TCA AAG TTA ACT TCA AAA CCA GAC ACA ACA TTG AAG ATG GAA GCG TTC AAC TAG CAG ACC ATT ATC AAC AAA ATA CTC CAA TTG GCG ATG GCC CTG TCC TTT TAC CAG ACA ACC ATT ACC TGT CCA CAC AAT CTG CCC TTT CGA AAG ATC CCA ACG AAA AGA GAG ACC ACA TGG TCC TTC TTG AGT TTG TAA CAG CTG CTG GGA TTA CAC ATG GCA TGG ATG AAC

Mm_bGluc 16 forward in pIB/V5-His-TOPO®

TTT AAT ACG ACT CAC TAT AGG GAG AAC AGT ATG GCA CCT TTG GCA AAT CAA CCG GGA GTT GGT GGT TAT ATC TGT AGT CGA ACT TTA TTA GTG GCC CAT GCT AAA GCT TAT CAT CTC TAC AAT GAT CGT TAC AAG GGA AGT CAA GGA GGT CGA GTG GGT ATT ACT ATA AAC AAC TTT TGG TAT GAA CCT TAT GCT GAT ACT ACA GAC TCA GCA GTA GAG TTA GCA TTA CAG GTT GCG TTT GGT TGG TTT ACT CAT CCC ATA TAT TCT AAA GAA GGC GAT TTC CCA CCA GCC ATG AAA GAA AGA ATA GCT AGA A

Mm_bGluc 17 reverse in pIB/V5-His-TOPO®

TTT AAT ACG ACT CAC TAT AGG GAG ATC TAG CTA TTC TTT CTT TCA TGG CTG GTG GGA AAT CGC CTT CTT TAG AAT ATA TGG GAT GAG TAA ACC AAC CAA ACG CAA CCT GTA ATG CTA ACT CTA CTG CTG AGT CTG TAG TAT CAG CAT AAG GTT CAT ACC AAA AGT TGT TTA TAG TAA TAC CCA CTC GAC CTC CTT GAC TTC CCT TGT AAC GAT CAT TGT AGA GAT GAT AAG CTT TAG CAT GGG CCA CTA ATA AAG TTC GAC TAC AGA TAT AAC CAC CAA CTC CCG GTT GAT TTG CCA AAG GTG CCA TAC TGT A

Mm_bGluc 17 forward in pIB/V5-His-TOPO®

TTT AAT ACG ACT CAC TAT AGG GAG AAC CCA CTC CCA TTT GTA TTG GAG GTT ATT CGG AGG GTT GGA TGG CGC CGG CTT ATG AAT TAC AAG GTG TTG GAG GTT ACC TGT GTG GTC ATA CAT TAC TTA TTG CTC ATG GGA AAG CTT ATC GGC TTT ACA ACG AAA AGT ATA GAG ATA CTC AGG AAG GTA GAG TTG GTA TAA CAA TTG ACA GCG GTT GGT ATG AAC CTG CTT CAG AAT CAG AAG AAG ACA TTC AAG CAG CTG AAG ACA GTG TAC ATA TAA AGT ACG GTT GGA TGG TCC ATC CGA TCT ATT CAG AGA CCG GTG ATT ATC CCC CTG TAT TGA GAG AAA GGG TAG ACG CAT TGA GTG CTG AAG AAG GCT TTG CGA GGT CCA GAT TGC CTA TCA

Mm_bGluc 17 reverse in pIB/V5-His-TOPO®

TTT AAT ACG ACT CAC TAT AGG GAG AGA TAG GCA ATC TGG ACC TCG CAA AGC CTT CTT CAG CAC TCA ATG CGT CTA CCC TTT CTC TCA ATA CAG GGG GAT AAT CAC CGG TCT CTG AAT AGA TCG GAT GGA CCA TCC AAC CGT ACT TTA TAT GTA CAC TGT CTT CAG CTG CTT GAA TGT CTT CTT CTG ATT CTG AAG CAG GTT CAT ACC AAC CGC TGT CAA TTG TTA TAC CAA CTC TAC CTT CCT GAG TAT CTC TAT ACT TTT CGT TGT AAA GCC GAT AAG CTT TCC CAT GAG CAA TAA GTA ATG TAT GAC CAC ACA GGT AAC CTC CAA CAC CTT GTA ATT CAT AAG CCG GCG CCA TCC AAC CCT CCG AAT AAC CTC CAA TAC AAA TGG GAG TGG GTA

Mm_bGluc 18 forward in pIB/V5-His-TOPO®

TTT AAT ACG ACT CAC TAT AGG GAG ATT CAT ATA TTT TGT GAA CTT GGA CAT TCC ATG GAT ATT ATG GCA CCG GCT CTT GGA TTG AGC GGA GTT GGT GGT TAT TTA TGC GCC CAT AAT ATA TTA ATA GCT CAT GGA AAG ACT TAT AAA TTG TAC AAT GAG AAG TAC AGG GAT ATT CAG AAA GGT AGA ATT GGT ATC ACA ATT GAT GGT GAA TGG AAA GAA CCG GCA TCC ACT TGT CCT GAA GAT ATA GAA ACG GCA GAA CGA GCT CTA CAA ATG GAG TTT GGT TGG ATA GCG CAC CCA ATC TTC TCA GAA TCC GGC GAT TAT CCA CCC GTT ATG AGG AGC AGA ATT GAC GTG ATG AGT GCC GAA GAA GGT CTG AAG ACA

Mm_bGluc 18 reverse in pIB/V5-His-TOPO®

TTT AAT ACG ACT CAC TAT AGG GAG AGT CTT CAG ACC TTC TTC GGC ACT CAT CAC GTC AAT TCT GCT CCT CAT AAC GGG TGG ATA ATC GCC GGA TTC TGA GAA GAT TGG GTG CGC TAT CCA ACC AAA CTC CAT TTG TAG AGC TCG TTC TGC CGT TTC TAT ATC TTC AGG ACA AGT GGA TGC CGG TTC TTT CCA TTC ACC ATC AAT TGT GAT ACC AAT TCT ACC TTT CTG AAT ATC CCT GTA CTT CTC ATT GTA CAA TTT ATA AGT CTT TCC ATG AGC TAT TAA TAT ATT ATG GGC GCA TAA ATA ACC ACC AAC TCC GCT CAA TCC AAG AGC CGG TGC CAT AAT ATC CAT GGA ATG TCC AAG TTC ACA AAA TAT ATG AAA
